# Supplementary material for: Marked isotopic variability within and between the Amazon River and marine dissolved black carbon pools
Source: Nat Commun. 2019 Sep 5;10:4018. doi: 10.1038/s41467-019-11543-9 (PMC6728373; doi:10.1038/s41467-019-11543-9)
Supplement: Supplementary file 1 — Supplementary Info [file 41467_2019_11543_MOESM1_ESM.pdf]

Supplementary Material for

Marked isotopic variability within and between the Amazon River and marine dissolved black carbon pools

Alysha I. Coppola[1*], Michael Seidel[2], Nicholas D. Ward[3-4], Daniel Viviroli[1], Negar Haghipour[5,6], Gabriela S. Nascimento[5,1], Brandi N. Revels[5], Samuel Abiven[1], Matthew W. Jones[7], Jeffery E. Richey[4], Timothy I. Eglinton[5], Thorsten Dittmar[2,8], Michael W.I. Schmidt[1]

[*]Corresponding author, email: Alysha.coppola@geo.uzh.ch

[1] Department of Geography, University of Zurich, Winterthurerstrasse 190, 8057 Zürich Switzerland

[2]Research Group for Marine Geochemistry, Institute for Chemistry and Biology of the Marine Environment (ICBM), University of Oldenburg, D-26129 Oldenburg, Germany

[3]Marine Sciences Laboratory, Pacific Northwest National Laboratory, 1529 West Sequim Bay Road, Sequim, Washington 98382 USA

[4]School of Oceanography, University of Washington, Box 355351 Seattle Washington 98195 USA

[5]Geological Institute, Department of Earth Sciences, ETH Zürich, Sonneggstrasse 5, 8092 Zürich Switzerland

[6]Laboratory of Ion Beam Physics, ETH Zürich, Otto-Stern-Weg 5, 8093 Zürich, Switzerland

[7]Tyndall Centre for Climate Change Research, University of East Anglia, Norwich NR4 7TJ, UK.

[8] Helmholtz Institute for Functional Marine Biodiversity at the University of Oldenburg (HIFMB) Ammerländer Heerstraße 231, 26129 Oldenburg, Germany

Supplementary Figures

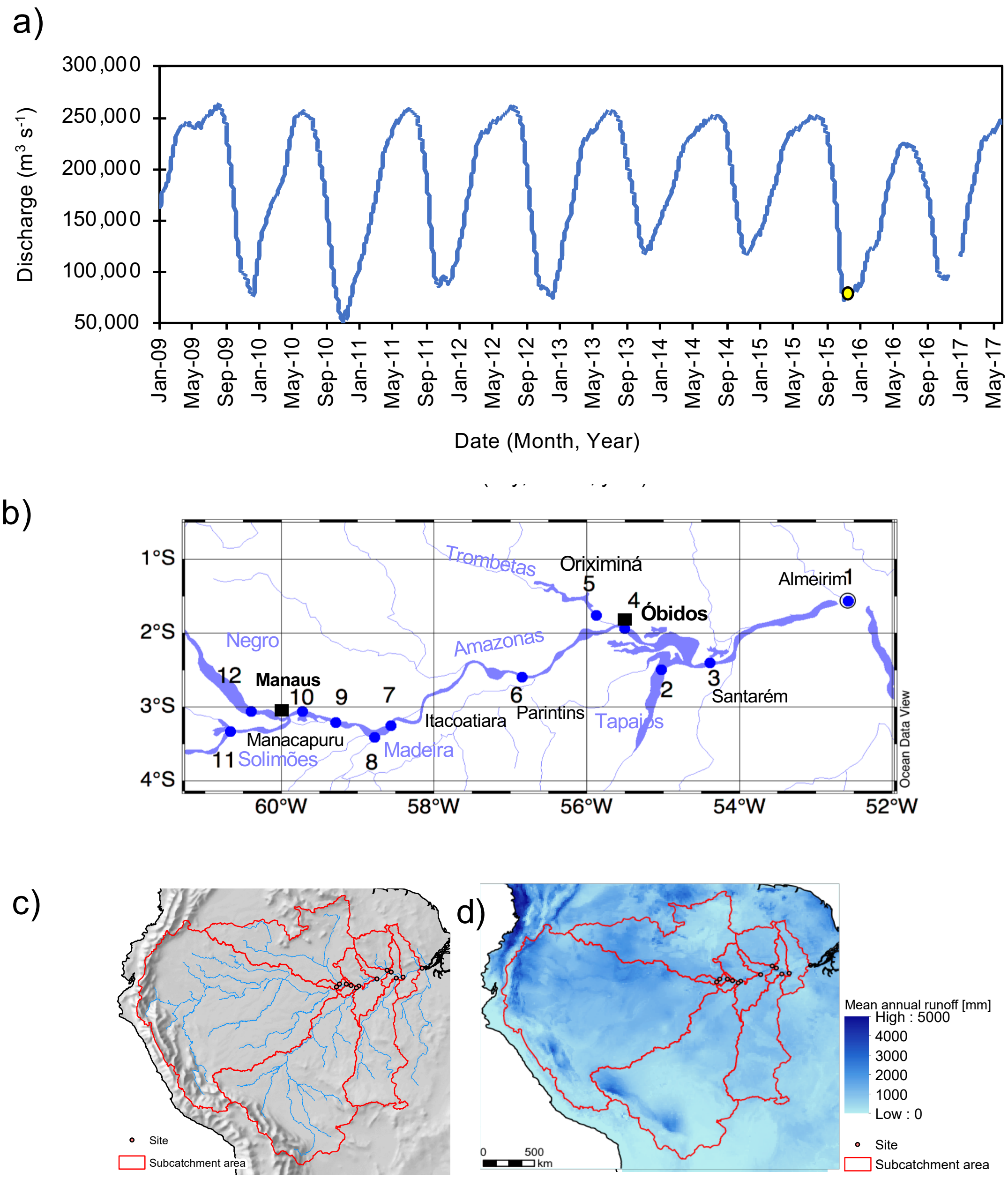

Supplementary Figure 1. a) Discharge time series from http://www3.ana.gov.br/portal/ANA/portal-ingles at Óbidos from 2009-2017, highlighting (yellow circle) the dry season sampling period in November 2015 following the El Nino drought. b) Map of the Amazon River and the major tributaries (in blue). Cities are shown in bold (black square). Numbered sample stations correspond to sampling locations in Supplementary Table 1. c) Corresponding sub-catchment areas (in red outlines). Catchment boundaries corresponding to each sample site were determined using a high-resolution (~500 m) stream

drainage direction map for the Amazon River basin [1]. Within these catchment boundaries, d) shows the average annual runoff integrated over the catchment area specific to sample sites 1-12 used in this study using a global map of streamflow characteristics based on observations (GSCD, Global Streamflow Characteristics Dataset)[2]. Figures c) and d) are created with ArcMap 10.6, relief shade from Natural Earth dataset (http://www.naturalearthdata.com), catchments derived from CAMREX (Carbon in the Amazon River Experiment) dataset[3] (https://daac.ornl.gov/LBA/guides/CD06_CAMREX.html), runoff from GSCD[2] (https://water.jrc.ec.europa.eu/).

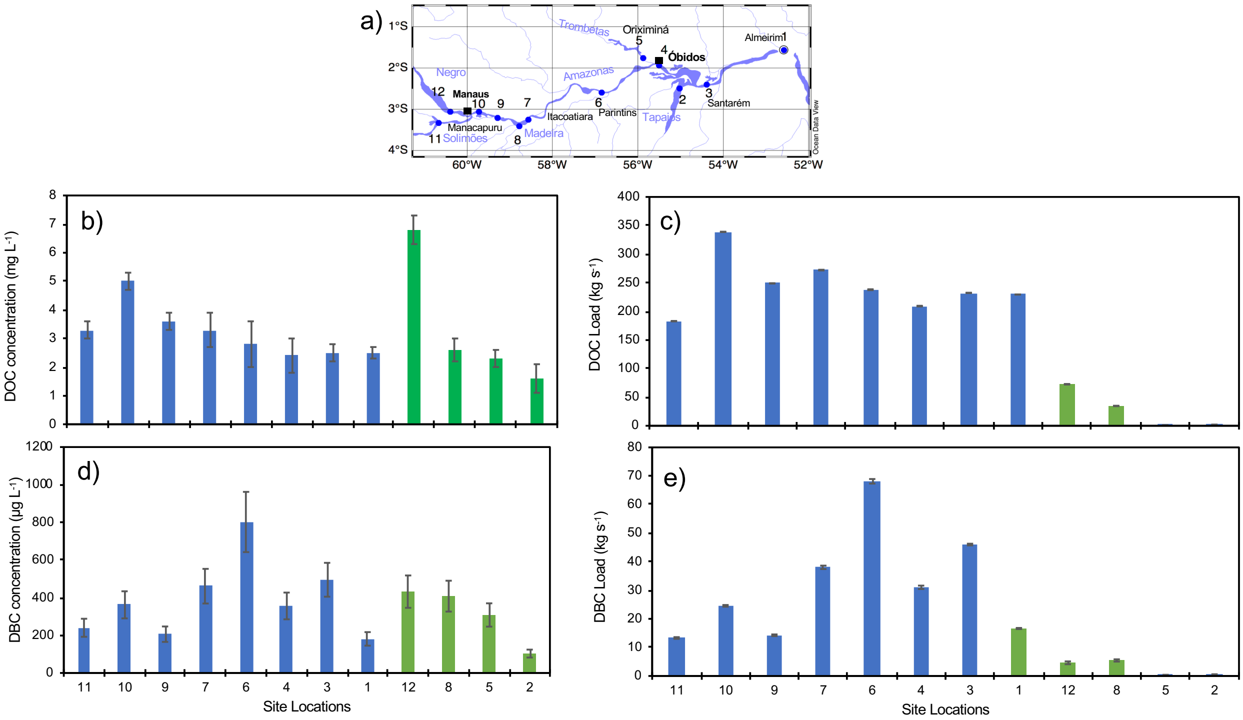

Supplementary Figure 2. a) Numbered site locations used in this study (see Supplementary Table 1). The concentrations and fluxes of at stations along the mainstem are shown in blue, and tributaries are shown in green. Error bars are determined from multiple measurements (n=3, s.d.). b) Dissolved Organic Carbon (DOC) concentration c) Dissolved Black Carbon (DBC) concentration d) DOC loads and e) DBC loads corresponding to site locations from upstream to downstream.

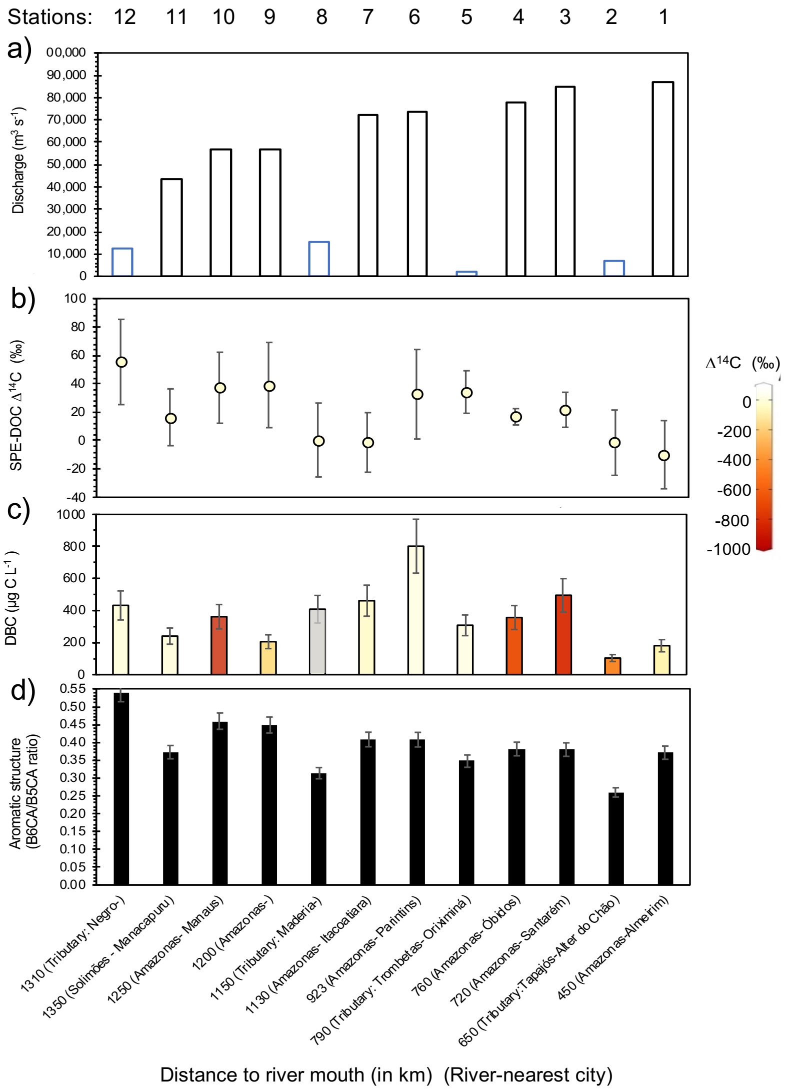

Supplementary Figure 3. Sample locations for this study (Supplementary Figure 1, Supplementary Table 1) plotted as distance to the river mouth (in km) with the river and nearest city in brackets for b-d sub plots. Station numbers are listed on top of the figure. Error bars are given by the internal error of $\Delta^{14}C$ measurements and the propagated error given by the processing of standards. Error bars for Dissolved Black Carbon (DBC) and aromatic structure are given by multiple measurements (n=3, s.d). a) Discharge, with blue bars representing discharge from tributaries. b) Solid phase extracted Dissolved Organic Carbon (SPE-DOC) $\Delta^{14}C$ values. The color represents modern $\Delta^{14}C$ values of Dissolved Organic Carbon (DOC). c) Dissolved Black Carbon (DBC) concentrations in μg DBC L^{-1} and DBC radiocarbon ($\Delta^{14}C$) values (given by the shade of red). The color grey on Station 8 represents no $\Delta^{14}C$ data, and d) the aromaticity of DBC given by the ratio of benzene polycarboxylic acid marker compounds with 5 and 6 carboxylic acid side groups (B6CA/B5CA). The higher the ratio, the greater the aromaticity/condensed structure of BC.

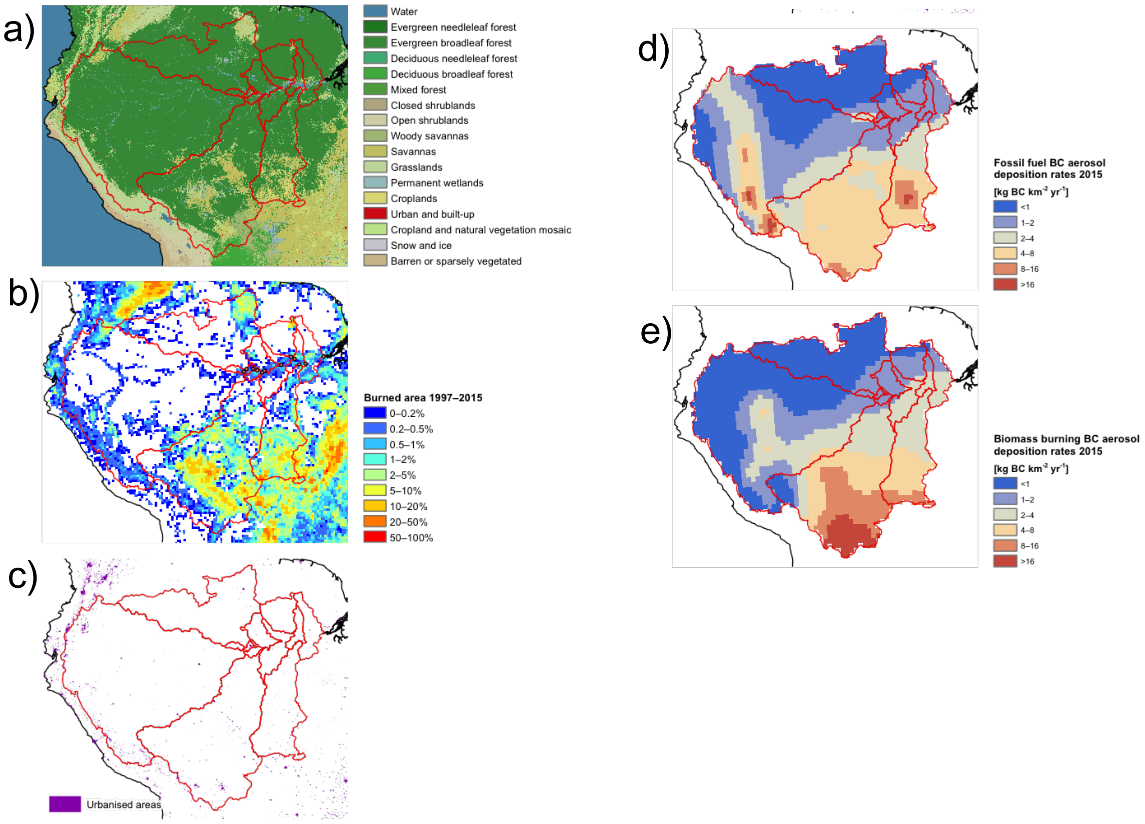

Supplementary Figure 4. Corresponding sub-catchment areas (in red outlines) for all sites were integrated over the catchment area specific to sample sites 1-12 used in this study. (See Methods Land use, urbanization and fire history). Catchment boundaries corresponding to each sample site were determined using a high-resolution (~500 m) stream drainage direction map for the Amazon River basin[1] a) Land cover from MODIS (Moderate Resolution Imaging Spectroradiometer, https://modis.gsfc.nasa.gov/data/dataprod/mod12.php) b) fraction of burned area from 1997- 2015, where white represents no forest fires burned during the time period from the Global Fire Emissions Database (GFED4s)[4] c) urbanized area (Global Rural-Urban Mapping Project GRUMP)[5], d) fossil fuel derived black carbon deposition rates in only 2015 in kg Black Carbon (BC) km^{-2} yr^{-1} and e) biomass BC deposition rates in only 2015 in kg BC km^{-2} yr^{-1} (See Methods Atmospheric Deposition of Black Carbon Aerosols). Values for the different parameters in the sampling catchments are listed in Supplementary Table 3. All figures were created with ArcMap 10.6, relief shade from Natural Earth dataset (http://www.naturalearthdata.com), catchments derived from CAMREX (Carbon in the Amazon River Experiment) dataset[3](https://daac.ornl.gov/LBA/guides/CD06_CAMREX.html).

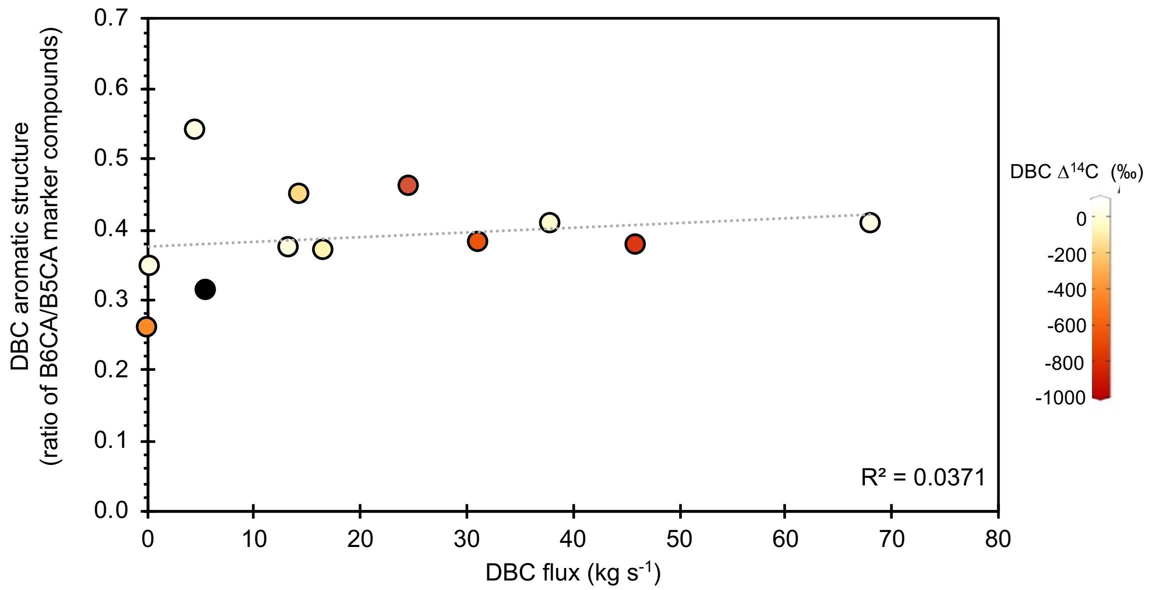

Supplementary Figure 5. No relationship between the aromatic Dissolved Black Carbon (DBC) aromatic structure (given by the ratio of Benzene Polycarboxylic Acids B6CA/B5CA marker compounds), DBC $\Delta^{14}C$ values (shades of red, solid black represents no DBC $\Delta^{14}C$ data) and DBC fluxes.

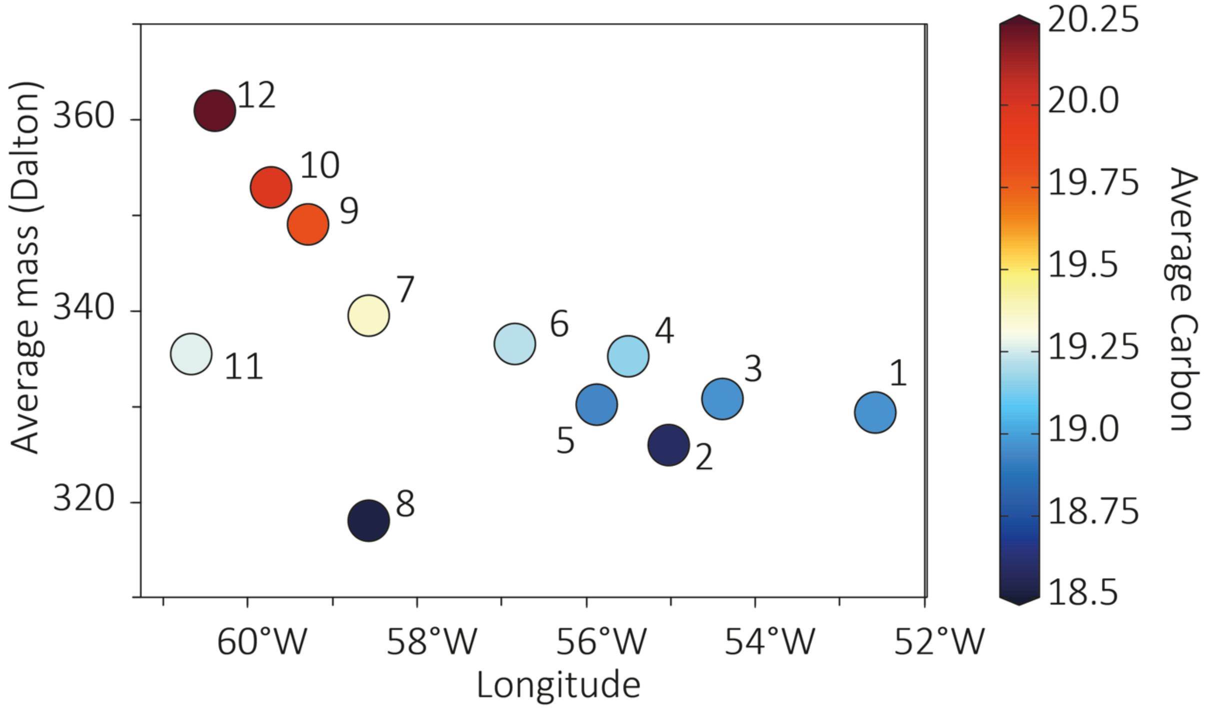

Supplementary Figure 6. Molecular parameters of the 1272 detected polycyclic aromatic molecular formulae with aromaticity index, AI_{mod} ≥0.67 as analyzed by Fourier transform ion cyclotron resonance mass spectrometry (FT-ICR-MS). Site codes refer to sample locations in Supplementary Table 1 from upstream to downstream. By definition, polycyclic aromatics include thermogenic Dissolved Black Carbon (DBC) molecular formulae. The intensity weighted-average molecular mass (in Daltons) of polycyclic aromatic molecular formulae over the sampling transect with color-coded weighted-average carbon numbers. Molecular masses and carbon numbers decreased downstream indicating the relative decrease of the size of polycyclic aromatic molecular formulae downstream.

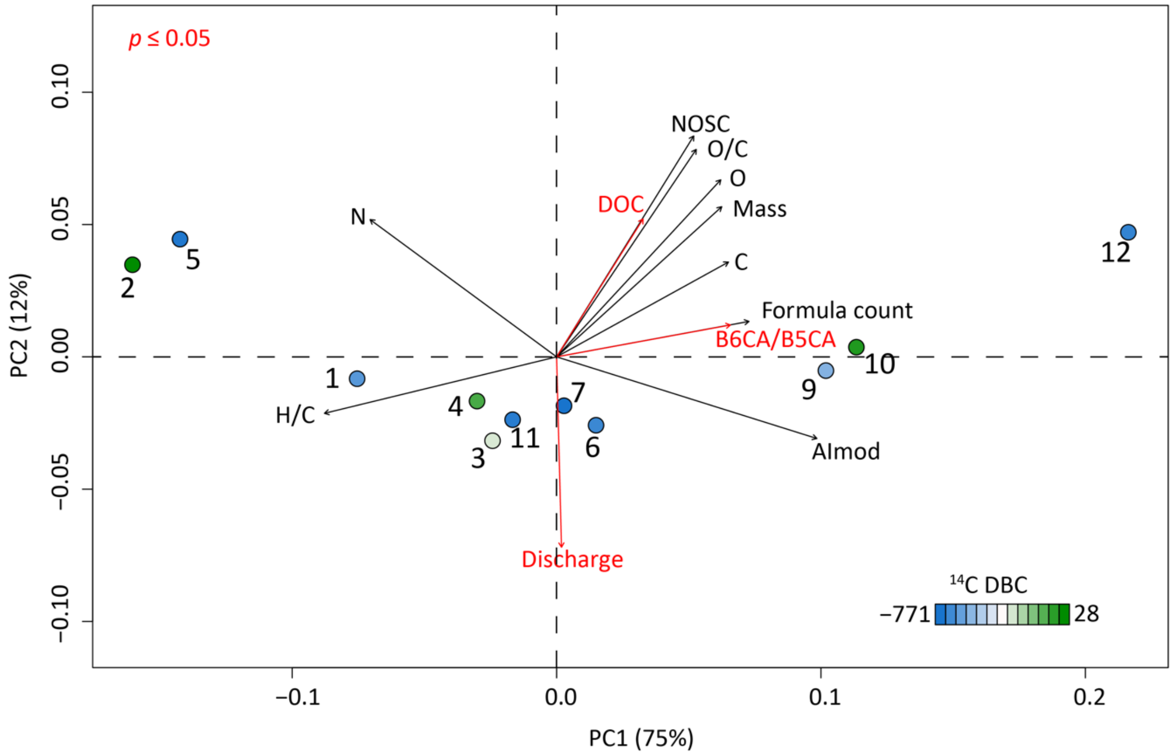

Supplementary Figure 7. Principal coordinate (PC) analyses based on Bray-Curtis dissimilarities of the relative abundance of polycyclic aromatic Dissolved Organic Matter (DOM) molecular formulae (aromaticity index, >0.67). The percentages give the polycyclic aromatic molecular variability as explained by the axes. Environmental parameters (red, significantly correlated with $p \leq 0.05$), intensity weighted averages of DOM molecular parameters (black, significant with $p < 0.01$), i.e., Oxygen/Carbon or Hydrogen/Carbon ratios, masses, Nitrogen, Carbon, Oxygen, nominal oxidation state (NOSC), Dissolved Organic Carbon (DOC), Benzene Polycarboxylic Acid ratios (as an indication of aromaticity B6CA/B5CA) and average number of molecular formulae per sample which were fitted onto the ordination. The color gradient shows $\Delta^{14}C$ of Dissolved Black Carbon (DBC) (‰) per station. Stations are given by the numbers corresponding to Supplementary Table 1.

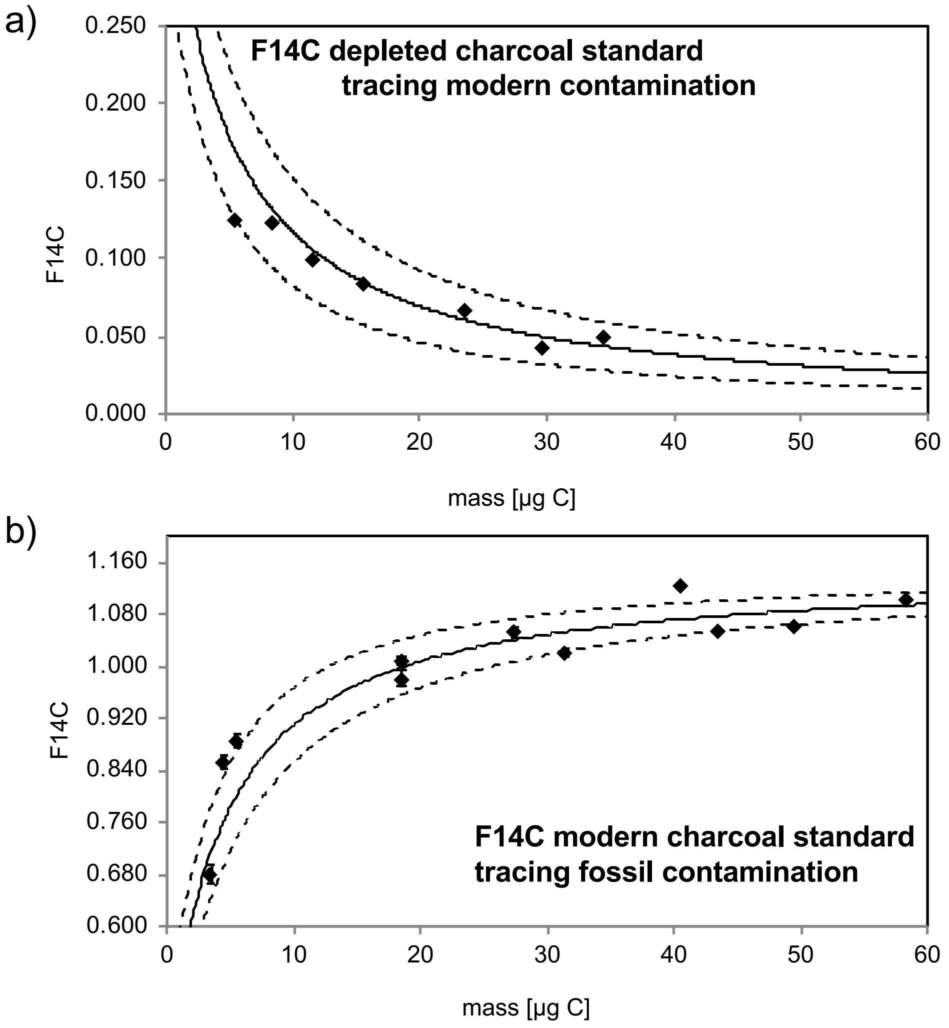

Supplementary Figure 8. a) Dead ($F^{14}C$=0.003±0.001) and b) modern ($F^{14}C$=1.149±0.004) wood char standards during the entire BPCA procedure were used to evaluate the extraneous, or blank carbon added to samples during chemical processing [6]. This reveals a constant contamination due to chemical processing.

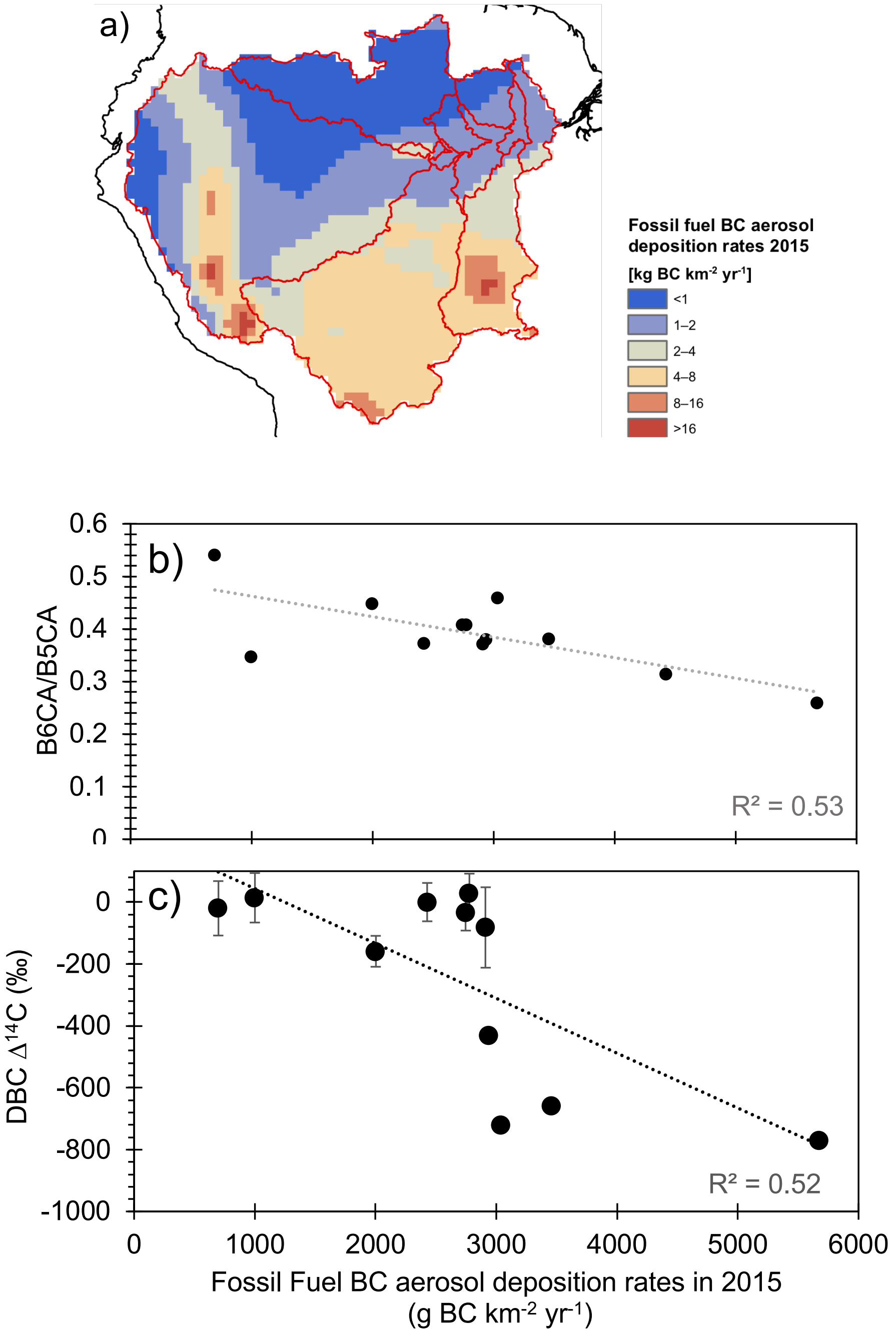

Supplementary Figure 9. a) Aerosol Black Carbon (BC) deposition was modelled in the Amazon River basin using the UK Met Office Hadley Centre Global Environment Model version 2 earth system model (HadGEM2-ES) (See Methods, Atmospheric Deposition of Black Carbon Aerosols). Figure 9a was created with ArcMap 10.6, relief shade from Natural Earth dataset (http://www.naturalearthdata.com), catchments derived from CAMREX (Carbon in the Amazon River Experiment) dataset[3]. Figure 9c) shows fossil fuel derived BC deposition integrated in corresponding sub-catchments per site and b) Benzene Polycarboxylic Acid

ratios B6CA/B5CA (r^2=0.53, $p<0.005$) and c) Dissolved Black Carbon (DBC) radiocarbon ($\Delta^{14}C$) values (r^2=0.52, $p<0.005$) (See Supplementary Discussion).

Supplementary Tables

Supplementary Table 1. Sample locations, station, descriptions, discharge and DOC characteristics.

Station	Sample ID	Latitude	Longitude	River	Catchment area [km²]	City	Distance	Collection date	Discharge Q [m3/s]	DOC [mg/L]	DOC [±mg/L]	DOC Load [kg/s]	SPE-DOC Δ14C [‰]	SPE-DOC [±‰]	SPE-DOC 13C [‰]	SPE-DOC [±‰]
11	MAC1.1N	3°19'45.7"	60°39'50.6"	Solimões	2,220,255	Manacapuru	1350	26.Nov.15	55,400	3.3	0.3	183	16	20	-30	0.1
10	MAN3.1	3°03'22.7"	59°43'10.0"	Amazonas	2,954,512	_____	1250	25.Nov.15	67,794	5.0	0.3	339	37	25	-29.4	0.1
9	MAN1.1	3°12'36.2"	59°16'20.9"	Amazonas	2,955,455	_____	1200	24.Nov.15	69,150	3.6	0.3	249	39	30	-30.2	0
7	IT1.1m	3°14'43.2"	58°34'12.5"	Amazonas	4,351,304	Itacoatiara	1130	23.Nov.15	82,467	3.3	0.6	272	-1	21	-31.5	0.1
6	PAR1.1m	2°35'33.4"	56°50'58.8"	Amazonas	4,431,204	Parintins	923	21.Nov.15	84,941	2.8	0.8	238	33	32	-29.8	0.1
4	OB1.1	1°55'59.7"	55°30'09.8"	Amazonas	4,678,899	Óbidos	760	18.Nov.15	87,149	2.4	0.6	209	17	6	-29.7	0
3	AM1.1	2°23'53.0"	54°23'51.0"	Amazonas	5,212,164	Santarém	720	17.Nov.15	92,914	2.5	0.3	232	22	12	-29.6	0.1
1	ALM1.1	1°33'41.9"	52°34'55.9"	Amazonas	5,328,347	Almeirim	450	16.Nov.15	91,768	2.5	0.2	229	-10	24	-29.1	0.2
12	tributary-NEG1.1N	3°02'57.5"	60°23'20.3"	Negro	714,021	_____	1310	27.Nov.15	10,673	6.8	0.5	73	55	30	-29.8	0.1
8	tributary-MAD1.1m	3°24'34.0"	58°47'03.5"	Madeira	1,378,622	_____	1150	23.Nov.15	13,318	2.6	0.4	35	0	26	-28.5	0.1
5	tributary-TP1.1	1°44'58.5"	55°52'49.1"	Trombetas	128,698	Oriximiná	790	19.Nov.15	400	2.3	0.3	1	34	15	-30.5	0.1
2	tributary-TAP1.1	2°29'44.9"	55°02'02.6"	Tapajós	485,803	Alter do Chão	650	17.Nov.15	1,000	1.6	0.5	2	-2	23	-29.2	0.1

Supplementary Table 2. Sampling stations, DBC amounts and DBC 14C values and DBC structural quality given by the relative abundance of BPCA marker compounds.

Station	Sample ID	River	DBC Concentration [ug/L]	DBC Concentration [± ug/L]	DBC Load [kg/s]	uncorrected DBC [F14C]	[±]	DBC Δ14C [‰]	DBC Δ14C [± ‰]	DBC F14C [F14C]	DBC F14C [±]	B6CA (%)	B5CA (%)	B4CA (%)	B3CA (%)	B6CA/B5CA (ratio)
11	MAC1.1N	Solimões	240	48	13.3	0.888	0.007	0	52	1.000	0.052	17.1	46.0	31.1	5.9	0.37
10	MAN3.1	Amazonas	362	72	24.5	0.288	0.004	-720	8	0.280	0.008	20.8	45.2	28.5	5.5	0.46
9	MAN1.1	Amazonas	206	41	14.2	0.751	0.007	-159	45	0.841	0.045	20.4	45.5	28.6	5.5	0.45
7	IT1.1m	Amazonas	461	92	38.0	0.945	0.008	28	54	1.028	0.054	18.8	46.0	29.5	5.7	0.41
6	PAR1.1m	Amazonas	802	160	68.1	0.861	0.007	-33	49	0.967	0.049	18.8	46.2	29.2	5.8	0.41
4	OB1.1	Amazonas	356	71	31.0	0.344	0.005	-658	7	0.342	0.007	17.6	46.2	30.3	5.9	0.38
3	AM1.1	Amazonas	495	90	46.0	0.548	0.006	-431	14	0.569	0.014	17.7	46.7	30.7	4.8	0.38
1	ALM1.1	Amazonas	181	36	16.6	0.745	0.007	-82	98	0.918	0.098	17.3	46.5	30.0	6.3	0.37
12	tributary-NEG1.1N	Negro	432	86	4.6	0.838	0.007	-20	70	0.980	0.071	23.7	43.9	27.0	5.4	0.54
8	tributary-MAD1.1m	Madeira	408	82	5.4			n.d.	n.d	n.d.		15.0	47.9	31.1	6.0	0.31
5	tributary-TP1.1	Trombetas	308	62	0.1	0.878	0.008	14	65	1.014	0.065	15.7	45.4	32.0	6.9	0.35
2	tributary-TAP1.1	Tapajós	103	21	0.1	0.250	0.004	-771	16	0.229	0.016	12.2	46.8	33.3	7.7	0.26

Supplementary Table 3. Land use and aerosol deposition contributions corresponding to Supplementary Figure 4.

		Land use (%) in the catchment corresponding to the sample site												Aerosol BC contributions	
station	Urbanization	Water	Evergreen Needleleaf forest	Evergreen Broadleaf forest	Deciduous Broadleaf forest	Mixed forest	Open shrublands	Woody savannas	Savannas	Grasslands	Permanent wetlands	Croplands	Cropland/Natural vegetation mosaic	Fossil fuel derived BC 2015 (g km-2 yr-1)	Biomass BC (g km-2 yr-1)
1	0.4	7%	0%	84%	0%	0%	0%	0%	4%	0%	2%	0%	3%	2912	3710
2	0.6	0%	0%	71%	0%	0%	0%	0%	21%	0%	0%	8%	0%	5676	5160
3	0.4	8%	0%	84%	0%	0%	0%	0%	5%	0%	2%	0%	1%	2940	3744
4	0.3	5%	0%	92%	0%	0%	0%	0%	0%	0%	3%	0%	1%	3459	3613
5	0.1	0%	0%	97%	0%	0%	0%	2%	0%	0%	0%	0%	1%	1000	1281
6	0.1	5%	0%	93%	0%	0%	0%	0%	0%	0%	2%	0%	0%	2747	3714
7	0.2	3%	0%	86%	0%	0%	0%	0%	0%	0%	9%	0%	1%	2777	3755
8	0.7	0%	0%	68%	3%	0%	1%	6%	14%	6%	1%	1%	0%	4425	9337
9	7.5	9%	0%	77%	0%	0%	0%	0%	0%	0%	2%	0%	5%	2001	1108
10	1.3	3%	0%	95%	0%	0%	0%	0%	0%	0%	2%	0%	0%	3038	1108
11	0.8	0%	0%	90%	0%	0%	0%	0%	1%	8%	0%	0%	1%	2428	1283
12	0.1	0%	0%	92%	0%	0%	0%	0%	2%	5%	1%	0%	0%	697	555

Supplementary Note 1

In addition, we found a decrease of the average molecular mass and carbon number of polycyclic aromatic molecular formulae (Figure 2) which corresponds to a decrease of the polycyclic aromatic molecular size. The samples contained ca. 460 to 980 polycyclic aromatic molecular formulae (Figure 2), accounting for 6 to 9% of the total molecular formulae for each sample which is in the range of polycyclic aromatic molecular formulae in Amazon River samples downstream of Óbidos[7]. The number of polycyclic aromatic decreased downstream and they were particularly low in clearwater tributary of Tapajós (station 2) but also at Trombetas (station 5) and Madeira (station 8) (Supplementary Table 1). This may indicate enhanced photodegradation in the clearwater rivers with higher light penetrations compared to the mainstem. The proportion of combustion-derived polycyclic aromatic molecular formulae decreased down the river, which corresponded to B6CA but not with the total concentration of DBC. As expected, DBC $\Delta^{14}C$ was not correlated to polycyclic aromatic molecular formulae (Supplementary Figure 7). However, we find that the polycyclic aromatic molecular formulae were related with the B6CA/B5CA molecular markers, DOC concentration and discharge (Supplementary Figure 7).

Supplementary Discussion

We hypothesize that the presence of strongly ^{14}C-depleted riverine DBC values may be related to source changes within the catchment area. Specifically, local inputs of fossil-fuel derived BC ($\Delta^{14}C$=-1000‰) might influence river DBC $\Delta^{14}C$ values. Aerosol transport has been shown to make a large contribution (5-22%) to DBC river fluxes in major tropical and temperate catchments[8,9]. Fossil fuel derived BC deposition rates increases with proximity to major cities (e.g. São Paulo and Rio de Janeiro,[10] but can also have local pollution regional influences (e.g. Manaus paired with prevailing easterly wind direction modulated by a significant river breeze)[11]. BC oxidation, photo-degradation and mixing with hydrophilic aerosols (e.g. sulphates and organic carbon) in aerosols and rainwater[12,13] can enhance its water solubility[14,15] during transport to rivers from the landscape over short timescales (1-2 days)[8].

Thus, greater inputs of fossil-fuel derived DBC ($\Delta^{14}C$=-1000‰) deposited over the integrated catchment area per site (0.5° resolution, Supplementary Figure 9) may decrease the modern river DBC isotopic composition. While DBC $\Delta^{14}C$ values were not related to land cover (p=0.385 for wetlands, p=0.094 for croplands, p=0.516 for natural vegetation) burned (p=0.283) or urbanized area (p=0.985) (Supplementary Table 3, Supplementary Figure 4), a significant correlation was found between BC fossil fuel aerosols contributions (Methods, Deposition of BC Aerosols) over the sub-catchment area and riverine $\Delta^{14}C$ DBC values (Supplementary Table 3, $p<0.005$). Notably, older DBC ages (lower $\Delta^{14}C$ values) were correlated with higher rates of atmospheric (aerosol) deposition as opposed to enhanced inputs of aged BC from soils (Supplementary Figure 9). Sites with low $\Delta^{14}C$ values had relatively large fossil fuel-derived aerosol deposition in the sub catchments of 4.2±1.6 kg BC km^{-2} whereas modern DBC values correspond to sites that have relatively low inputs of fossil fuel derived BC (2.2±1.0 kg BC km^{-2}).

Despite the correlations that we observed between DBC aromatic structure and DBC $\Delta^{14}C$ with upstream fossil fuel aerosol deposition rates (Supplementary Figure 9), there is limited evidence that regional-scale aerosol deposition to Amazonia is sufficient to make single large contributions to the riverine DBC export flux. The annual rate of fossil fuel derived BC aerosol deposition to the Amazon catchment (0.016 to 0.032 Tg C $year^{-1}$) when integrated across the area upstream of Almeirim (Supplementary Table 3), is equivalent to ~1% of the annual DBC flux exported from the Amazon River (1.9-2.7 Tg C $year^{-1}$). For example, estimates of atmospheric fossil-fuel contributions (15-22%) to DBC in Chinese rivers based on a different method (or larger PBC ranging from 50-75% fossil fuel inputs)[9,16]. The use of a different method may underestimate the proportion of fossil fuel derived BC. In addition, Sun et al.,[17] suggest that BC emissions have been underestimated by 80% in North America, and it may be true for other locations. It is unlikely that $\Delta^{14}C$ DBC values are driven alone by regional fossil fuel aerosols. Nonetheless, this does not exclude the possibility that highly localized

emission and deposition of BC aerosol, occurring in urban centers and on waterways at sub-grid scales, could affect the load and age of DBC in our samples.

Overall, we suggest that the marked low DBC $\Delta^{14}C$ values may be driven by atmospheric deposition of fossil fuel derived BC from local point sources, superimposed upon a dominant DBC flux from modern sources. Considering that each mainstem station bares the quantitative legacy of everything upstream of that point, there must be a rapid process such as sorption or decomposition driving the downstream variability in DBC $\Delta^{14}C$ values. Thus, we hypothesize that the rapid shifts from surface low DBC $\Delta^{14}C$ values (from potentially fossil fuel aerosol impacted sites) to modern $\Delta^{14}C$ values at downstream sites (sampled at 1m water depth) may be rapidly removed by transferring to larger size particulate BC pools in both suspended and benthic sediments, thus removing fossil fuel signals from the dissolved phase[18]. Indeed, sorption and desorption is a rapid process that alters DOM composition along the Amazon River continuum[19]. One other possible explanation for low DBC $\Delta^{14}C$ values in mainstem sites just downstream of tributaries (e.g., Santarém) is that the collected water represented an impartial mixture of tributary and mainstem water considering that tributaries in the Amazon have been shown to be poorly mixed up to 100 km downstream of their confluence.[20]

Locally high contributions of fossil DBC to such a globally-significant and relatively pristine catchment would suggest that point sources of fossil fuel BC aerosol should be considered in ongoing assessments of BC and DOC dynamics in river systems. For example, there is an urban anthropogenic aerosol plume 8-70 km downwind of Manaus from regional urbanization[21] that could contribute to the low $\Delta^{14}C$ DBC values observed at site 10 (20km downwind)[11]. Locally high contributions of fossil DBC to such a globally-significant and relatively pristine catchment (Amazon basin) may suggest that fossil fuel-derived BC should be considered in assessments of contemporary – and future - BC and DOC cycling studies to

address this conundrum. Increases of local anthropogenic emissions in the future may enhance aerosol pollution in pristine areas like the Amazon[21].

Supplementary References

1 Mayorga, E., Logsdon, M. G., Ballester, M. V. R. & Richey J.E. (ed LBA-ECO CD-06) (Oak Ridge National Laboratory Distributed Active Archive Center, Oak Ridge, Tennessee, U.S.A., 2012).

2 Beck, H. E., Roo, A. d. & Dijk, A. I. J. M. v. Global Maps of Streamflow Characteristics Based on Observations from Several Thousand Catchments. *Journal of Hydrometeorology* **16**, 1478-1501, doi:10.1175/jhm-d-14-0155.1 (2015).

3 Mayorga, E., Logsdon, M. G., Ballester, M. V. R. & Richey, J. E. (ORNL Distributed Active Archive Center, 2012).

4 van der Werf, G. R. *et al.* Global fire emissions estimates during 1997–2016. *Earth Syst. Sci. Data* **9**, 697-720, doi:10.5194/essd-9-697-2017 (2017).

5 Center for International Earth Science Information Network - CIESIN - Columbia University, International Food Policy Research Institute - IFPRI, The World Bank & Centro Internacional de Agricultura Tropical - CIAT. (NASA Socioeconomic Data and Applications Center (SEDAC), Palisades, NY, 2017).

6 Hammes, K. *et al.* Comparison of quantification methods to measure fire-derived (black/elemental) carbon in soils and sediments using reference materials from soil, water, sediment and the atmosphere. *Global Biogeochemical Cycles* **21**, doi:10.1029/2006gb002914 (2007).

7 Seidel, M. *et al.* Seasonal and spatial variability of dissolved organic matter composition in the lower Amazon River. *Biogeochemistry* **131**, 281-302, doi:10.1007/s10533-016-0279-4 (2016).

8 Jones, M. W. *et al.* Do Regional Aerosols Contribute to the Riverine Export of Dissolved Black Carbon? *J. Geophys. Res.-Biogeosci.* **122**, 2925-2938, doi:10.1002/2017jg004126 (2017).

9 Wang, X., Xu, C., Druffel, E. M., Xue, Y. & Qi, Y. Two black carbon pools transported by the Changjiang and Huanghe Rivers in China. *Global Biogeochemical Cycles*, n/a-n/a, doi:10.1002/2016GB005509 (2016).

10 Jones, M. W. *et al.* Enviromental Controls on the Riverine Export of Dissolved Black Carbon. (accepted 2019).

11 Cirino, G. *et al.* Observations of Manaus urban plume evolution and interaction with biogenic emissions in GoAmazon 2014/5. *Atmospheric Environment* **191**, 513-524, doi:https://doi.org/10.1016/j.atmosenv.2018.08.031 (2018).

12 Maskey, S. *et al.* Cloud Condensation Nuclei Activation of Internally Mixed Black Carbon Particles. *Aerosol and Air Quality Research* **17**, 867-877, doi:10.4209/aaqr.2016.06.0229 (2017).

13 Textor, C. *et al.* Analysis and quantification of the diversities of aerosol life cycles within AeroCom. *Atmospheric Chemistry and Physics* **6**, 1777-1813, doi:10.5194/acp-6-1777-2006 (2006).

14 Mead, R. N. *et al.* Insights into dissolved organic matter complexity in rainwater from continental and coastal storms by ultrahigh resolution Fourier transform ion cyclotron resonance mass spectrometry. *Atmos. Chem. Phys.* **13**, 4829-4838, doi:10.5194/acp-13-4829-2013 (2013).

15 Decesari, S. *et al.* Water soluble organic compounds formed by oxidation of soot. *Atmospheric Environment* **36**, 1827-1832, doi:https://doi.org/10.1016/S1352-2310(02)00141-3 (2002).

16 Bao, H., Niggemann, J., Luo, L., Dittmar, T. & Kao, S.-J. Aerosols as a source of dissolved black carbon to the ocean. *Nature Communications* **8**, 510, doi:10.1038/s41467-017-00437-3 (2017).

17 Sun, T. *et al.* Constraining a Historical Black Carbon Emission Inventory of the United States for 1960–2000. *Journal of Geophysical Research: Atmospheres* **124**, 4004-4025, doi:10.1029/2018jd030201 (2019).

18 Park, E. & Latrubesse, E. M. The hydro-geomorphologic complexity of the lower Amazon River floodplain and hydrological connectivity assessed by remote sensing and field control. *Remote Sensing of Environment* **198**, 321-332, doi:https://doi.org/10.1016/j.rse.2017.06.021 (2017).

19 Aufdenkampe, A. K., Hedges, J. I., Richey, J. E., Krusche, A. V. & Llerena, C. A. Sorptive fractionation of dissolved organic nitrogen and amino acids onto fine sediments within the Amazon Basin. *Limnology and Oceanography* **46**, 1921-1935, doi:10.4319/lo.2001.46.8.1921 (2001).

20 Bouchez, J. *et al.* Turbulent mixing in the Amazon River: The isotopic memory of confluences. *Earth and Planetary Science Letters* **290**, 37-43, doi:https://doi.org/10.1016/j.epsl.2009.11.054 (2010).

21 Shrivastava, M. *et al.* Urban pollution greatly enhances formation of natural aerosols over the Amazon rainforest. *Nature Communications* **10**, 1046, doi:10.1038/s41467-019-08909-4 (2019).
